# Evaluation of *BRCA1/2* testing rates in epithelial ovarian cancer patients: lessons learned from real-world clinical data

**DOI:** 10.1007/s10689-025-00467-7

**Published:** 2025-05-05

**Authors:** Lieke Lanjouw, Claire J. H. Kramer, Arja ter Elst, Geertruida H. de Bock, Katja N. Gaarenstroom, Refika Yigit, Lieke P. V. Berger, Christi J. van Asperen, Sabrina Z. Commandeur-Jan, Dimas M. X. van der Hall, Mathilde Jalving, Marjolein J. Kagie, Nienke van der Stoep, Tom van Wezel, Marian J. E. Mourits, Tjalling Bosse, Joost Bart

**Affiliations:** 1 Department of Epidemiology, University of Groningen, University Medical Center Groningen, PO Box 30.001, 9700 RB, Groningen, The Netherlands; 2 Department of Pathology, Leiden University Medical Center, Leiden, The Netherlands; 3 Department of Pathology and Medical Biology, University of Groningen, University Medical Center Groningen, Groningen, The Netherlands; 4 Department of Gynecology, Leiden University Medical Center, Leiden, The Netherlands; 5 Department of Obstetrics and Gynecology, University of Groningen, University Medical Center Groningen, Groningen, The Netherlands; 6 Department of Genetics, University of Groningen, University Medical Center Groningen, Groningen, The Netherlands; 7 Department of Clinical Genetics, Leiden University Medical Center, Leiden, The Netherlands; 8 Department of Medical Oncology, University of Groningen, University Medical Center Groningen, Groningen, The Netherlands; 9 Department of Gynecology, Haaglanden Medisch Centrum, The Hague, The Netherlands

**Keywords:** Ovarian cancer, *BRCA1/2*, Tumor test, Genetic predisposition, Evaluation

## Abstract

Identification of somatic and germline *BRCA1/2* pathogenic variants in epithelial ovarian cancer (EOC) patients is essential for determining poly-(ADP-ribose)-polymerase (PARP) inhibitor sensitivity and genetic predisposition. In the Netherlands, *BRCA1/2* testing changed to a tumor-first approach to efficiently identify both somatic and germline pathogenic variants in all patients. Here, we performed an in-depth evaluation of the first four years of the tumor-first test-pathway. Data of consecutive series of patients diagnosed with EOC in two regions were obtained from the Netherlands Cancer Registry. Tumor and/or germline test data were retrieved from hospital databases. The primary outcome was the percentage of patients completing the *BRCA1/2* test-pathway, defined as having a negative tumor test or a referral for a germline test in case of a positive tumor test or no tumor test. Factors associated with test-pathway completion were identified through multivariable logistic regression analysis. In total, 69.8% (757/1085) completed the test-pathway. This was 74.4% in the most recent year. Younger patients, patients diagnosed in year three or four, patients with high-grade serous/high-grade endometrioid carcinoma, advanced stage disease, middle or high socioeconomic status, and patients who underwent surgery or chemotherapy, were more likely to complete the test-pathway. We report inequalities in genetic testing access in EOC patients, which highlight the need for better guideline adherence, particularly in older patients, those with low socioeconomic status, low-grade histotypes, early-stage disease and those without surgery or chemotherapy. Additionally, timely testing of patients, and testing relatives if patients cannot be tested, are crucial to increase test uptake.

**Supplementary Information:**

The online version contains supplementary material available at 10.1007/s10689-025-00467-7.

## Introduction

The importance of detecting germline *BRCA1/2* (likely) pathogenic variants (PVs) in epithelial tubal/ovarian cancer (EOC) patients to determine genetic predisposition has been well recognized. Beyond *BRCA1/2* PVs in the germline, the identification of somatic PVs in the tumor has become increasingly important, predominantly driven by promising results of poly-(ADP-ribose)-polymerase (PARP) inhibitors in EOC patients [1–3]. As a result, testing for both germline and somatic PVs has been endorsed by international guidelines for all EOC patients [4, 5]. In the Netherlands, this substantially challenged the, at the time, germline-based testing workflow in EOC patients. While previously a germline test was offered to all patients [6], from 2016 onwards, a tumor-based workflow was implemented. In this so-called ‘tumor-first’ approach, DNA isolated from tumor tissue is tested first. Subsequently, patients with PVs detected in the tumor, or those with a family history of EOC or breast cancer, are referred for a germline test [7]. A nationwide implementation project was initiated to ensure uniform execution and monitoring of the tumor-first workflow throughout the Netherlands [8].

The novel tumor-first workflow simultaneously identifies (i) eligible patients for PARP inhibitors and (ii) those who require further germline testing, both with a near-perfect sensitivity [9]. Since the initiation of this *BRCA1/2* testing workflow is tumor- rather than patient-based, this approach is considered more inclusive than germline-based approaches [10]. In approximately half of the EOC patients with a *BRCA1/2* PV detected in the tumor, the PV is of germline origin [11–13] and their relatives are invited for counseling and testing, regardless of the cancer history of the relatives. Identification of relatives carrying the same germline PV is crucial to reduce EOC and breast cancer incidence and mortality as they can take risk-reducing measures, including risk-reducing salpingo-oophorectomy, breast screening or risk-reducing mastectomy.

We have previously demonstrated in a single-center study that not every EOC patient received the recommended testing, and that testing rates of the germline-first approach were comparable to those of the tumor-first approach in the early implementation phase [14]. Preliminary results from the Dutch implementation project showed promising tumor testing rates of 80% but lack insight into the proportion of patients completing the test-pathway, including germline test referrals if required, and characteristics of the patient populations that did not receive the recommended testing [8]. Characterizing these patients can help identify barriers in the current workflow, which is essential to further optimize testing rates. Therefore, we conducted an in-depth, patient-level evaluation of the first four years of the tumor-first workflow in EOC patients diagnosed in two large regions in the Netherlands.

## Methods

### Study population

The current study analyzed the catchment areas of two specialized gynecologic oncology university hospitals, referred to as region A and B, each serving approximately 1.7 million inhabitants. In addition to one specialized university hospital in each region, region A and B include eight and five regional hospitals respectively. The study was approved by the ethics committees of University Medical Center Groningen and Leiden University Medical Center, and informed consent was waived (reference numbers: 202200330; nWMO-D4-2022-030).

Consecutive series of patients (≥ 18 years) diagnosed with EOC in region A or B were obtained from the Netherlands Cancer Registry [15]. Timeframes depended on implementation of the tumor-first workflow and a run-in of three months was considered to account for the early implementation phase. Patients in region A were included if diagnosed between October 2018 and October 2022, and in region B if diagnosed between January 2018 and January 2022. Patients who moved outside of the region during treatment, or whose treatment was relocated to another hospital outside of the region, were considered as being out of scope for the purposes of this evaluation and were excluded.

## Outcome definition

**Fig. 1:**
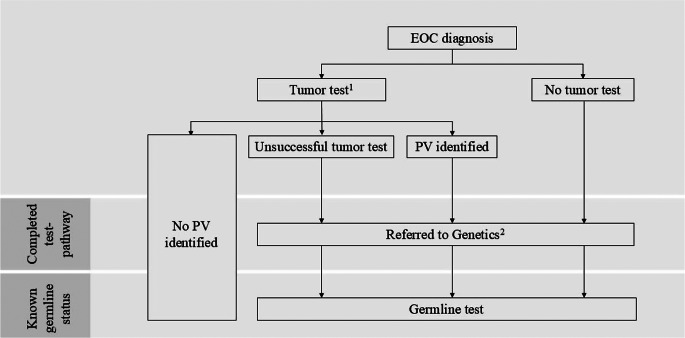
Flowchart of tumor-first workflow as evaluated in the current study For every patient with a negative tumor test (i.e., no pathogenic variant in the tumor), the test-pathway was considered to be completed, and the patient’s germline status was considered to be known (e.g., *BRCA1/2* wildtype). If the tumor test was positive (i.e., pathogenic variant in the tumor) or unsuccessful, it was evaluated whether the patient received a referral to Genetics, indicating the completion of the test-pathway, and if subsequently a germline test was performed, the germline status of the patient was considered to be known. ^1^ Requested by pathologist (if patient has not declined the test) and performed by laboratory specialist in specialized gynecologic oncology centers. ^2^ Gynecologist or medical oncologist refers patient if indicated. Referrals before EOC diagnosis and referrals for children of patient were included. Abbreviations: EOC, epithelial tubal/ovarian cancer; PV, pathogenic variant

The tumor-first workflow is visualized in Fig. 1. The primary outcome was the percentage of patients completing the test-pathway, defined as having (i) a negative tumor test (i.e., no PVs detected) or (ii) a referral for a germline test in case of a positive tumor test (i.e., PVs detected) or no tumor test. This included germline test referrals prior to diagnosis, e.g., following the detection of a PV in a family member. If a patient declined a germline test after being referred, the test-pathway was considered to be completed, regardless of tumor test outcome. Additionally, if it was reported in the patient files that children of the patient were referred for a germline test because it was not possible to test the patient herself, the test-pathway was also considered as being completed.

The secondary outcome was the percentage of patients with a known germline status, which was established in the following cases: (i) a tumor test confirming the absence of a germline or somatic PV, (ii) a germline test confirming the absence of a germline PV, or (iii) a germline test confirming the presence of a germline PV.

## Data collection

Data obtained from the Netherlands Cancer Registry included patient identification (ID) codes, date of diagnosis, histotype, International Federation of Gynecology and Obstetrics (FIGO) stage, performance status, comorbidities, socioeconomic status (SES), and type of treatment. Variable descriptions can be found in Supplementary Table 1.

Regional patient IDs were linked to corresponding IDs from the testing center using pathology databases containing records from all patients whose tissue has been analyzed for diagnostics and/or a tumor test. Since tumor testing is centralized in the university hospitals of both regions, it was assumed that patients whose tumor tissue was tested could be identified via this route. For patients who could not be identified at the testing center, regional hospitals were contacted to check whether there was information on tumor and/or germline tests. Databases of the testing centers were used to assess whether patients received *BRCA1/2* testing according to current national guidelines (Fig. 1). Data regarding *BRCA* status were available from the Netherlands Cancer Registry for a subset of the patients and used to supplement data from hospital databases. In both university hospitals (the testing centers) included in the current study, gene panels for the tumor test expanded over time from analysis of *BRCA1/2* only to also testing for *BRIP1*, *RAD51C*, *RAD51D* and *PALB2*, which is in line with the latest national guideline [7]. The applied sequencing techniques can vary between testing centers [16] and have been described previously for the two testing centers included [17].

### Data analysis

Patient and clinical characteristics were described for the overall cohort and by region. Continuous variables were reported as mean and standard deviation (SD) for normally distributed variables, and as median and interquartile range (IQR) for non-normally distributed variables. Categorical variables were reported as count (*n*) and percentage. The percentage of patients who completed the test-pathway was analyzed for all patients with the indication to test, as well as by region and year of the tumor-first approach.

Univariate and multivariable logistic regression analyses were performed to identify reasons for not completing the test-pathway. Variables included in the logistic regression analyses were variables that could influence test-pathway completion and were available for our cohort. These variables included year of diagnosis, age at diagnosis, region of diagnosis, histotype, FIGO stage, performance status, comorbidities, SES, surgery, and chemotherapy. Odds ratios (OR), 95% confidence intervals (CIs) and *p-*values were reported. Variables that were significantly (*p* < 0.05) associated with the outcome in univariate analysis, were included in the multivariable model. The analyses were performed using SPSS Statistics version 28 (IBM Corp, Armonk, NY, USA).

## RESULTS

A total of 1108 patients with EOC were identified, of whom 1085 patients had a test indication (Supplementary Fig. 1). Mean age of the patients was 70 years (IQR 60.0–77.0). Most patients were diagnosed with high-grade serous carcinoma (56.4%) and with FIGO stage III (46.5%) (Table 1).

**Table 1:** Cohort characteristics

Characteristics	Total (*N* = 1085) *n* (%)	Region A (*n* = 568) *n* (%)	Region B (*n* = 517) *n* (%)
Age, median (IQR)	70.0 (60.0–77.0)	69.0 (60.0–77.0)	70.0 (58.0–78.0)
Number of diagnoses, in TF years			
First year	269 (24.8)	145 (25.5)	124 (24.0)
Second year	293 (27.0)	154 (27.1)	139 (26.9)
Third year	273 (25.2)	139 (24.5)	134 (25.9)
Fourth year	250 (23.0)	130 (22.9)	120 (23.2)
Histotype			
High-grade serous	612 (56.4)	327 (57.6)	285 (55.1)
Endometrioid^1^	73 (6.7)	42 (7.4)	31 (6.0)
Low-grade serous	64 (5.9)	31 (5.5)	33 (6.4)
Clear cell	67 (6.2)	38 (6.7)	29 (5.6)
Mucinous	64 (5.9)	36 (6.3)	28 (5.4)
Carcinosarcoma	26 (2.4)	16 (2.8)	10 (1.9)
Other^2^	69 (6.4)	32 (5.6)	37 (7.2)
Adenocarcinoma NOS	110 (10.1)	46 (8.1)	64 (12.4)
FIGO stage			
I	176 (16.2)	93 (16.4)	83 (16.1)
II	71 (6.5)	37 (6.5)	34 (6.6)
III	505 (46.5)	253 (44.5)	252 (48.7)
IV	311 (28.7)	174 (30.6)	137 (26.5)
Unknown	22 (2.0)	11 (1.9)	11 (2.1)
Performance status			
0	289 (26.6)	177 (31.2)	112 (21.7)
1	189 (17.4)	118 (20.8)	71 (13.7)
2	61 (5.6)	41 (7.2)	20 (3.9)
3	29 (2.7)	18 (3.2)	11 (2.1)
4	8 (0.7)	4 (0.7)	4 (0.8)
Unknown	509 (46.9)	210 (36.9)	299 (57.8)
Comorbidities			
0	352 (32.4)	262 (46.1)	90 (17.4)
1–2	206 (19.0)	158 (27.8)	48 (9.3)
3–4	19 (1.8)	14 (2.5)	5 (1.0)
>5	1 (0.1)	.	1 (0.2)
Unknown	507 (46.7)	134 (23.6)	373 (72.1)
SES			
Low	242 (22.3)	132 (23.2)	110 (21.3)
Middle	482 (44.4)	281 (49.5)	201 (38.9)
High	241 (22.2)	87 (15.3)	154 (29.8)
Unclassifiable	120 (11.1)	68 (12.0)	52 (10.1)
Surgery			
Yes	744 (68.6)	386 (68.0)	358 (69.2)
No	341 (31.4)	182 (32.0)	159 (30.8)
Chemotherapy			
Yes	744 (68.6)	391 (68.8)	353 (68.3)
No	341 (31.4)	177 (31.2)	164 (31.7)

^1^ Including 26 high-grade endometrioid carcinomas; 41 low-grade endometrioid carcinomas, and 6 endometrioid carcinomas with unknown grade. ^2^ Including neuro-endocrine tumors, small-cell carcinoma of the ovary hypercalcemic type, and mesonephric adenocarcinoma. Abbreviations. IQR, interquartile range; TF, tumor-first; NOS, not otherwise specified; FIGO, Fédération Internationale de Gynécologie et d’Obstétrique; SES, socio-economic status

**Fig. 2:**
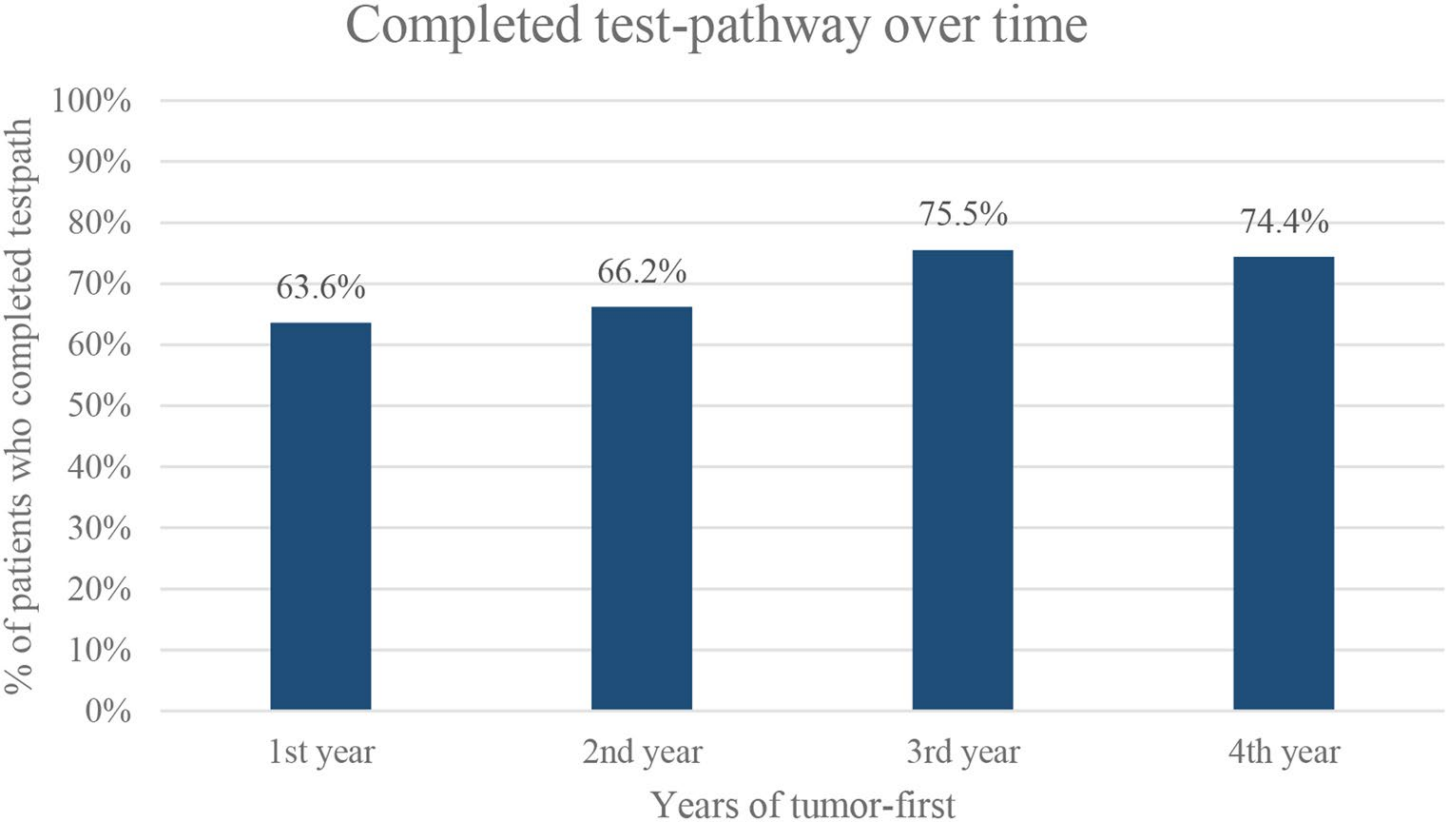
The percentage of patients who completed the *BRCA1/2* test-pathway, in years of the tumor-first workflow

The overall percentage of patients who completed the *BRCA1/2* test-pathway was 69.8% (Table 2). This percentage increased from 63.6% in the first year of the tumor-first approach to 74.4% in the fourth year (Fig. 2). The percentage of patients with a known germline status was 68.5% (Table 2). Results of region A and B were comparable (Supplementary Table 2).

**Table 2:** Outcomes of the *BRCA1/2* test-pathway evaluation in patients with indication for test, *n* (%)

	Patients with indication for test (*n* = 1085)
Completed test-pathway^1^	757 (69.8)
Known germline status	743 (68.5)

^1^ Includes 3 patients who passed away between referral and germline test

The following variables were significantly associated with completing the test-pathway and included in the multivariable model: age at diagnosis, year of diagnosis, FIGO stage, histotype, SES, performance status, comorbidities, surgery, and chemotherapy (Table 3). Older patients were less likely to complete the test-pathway (OR 0.89, 95% CI 0.83–0.96, *p* = 0.002). Patients diagnosed in the third or fourth year of the tumor-first approach were more likely to complete the test-pathway compared to those diagnosed in the first year (OR 2.52, 95% CI 1.52–4.20, *p* < 0.001; OR 3.65, 95% CI 2.11–6.30, *p* < 0.001, respectively). Patients diagnosed with FIGO stage III/IV and with high-grade serous/high-grade endometrioid carcinoma were more likely to complete the test-pathway compared to FIGO stage I/II and other histotypes, respectively (OR 2.10, 95% CI 1.24–3.53, *p* = 0.005; OR 2.86, 95% CI 1.96–4.18, *p* < 0.001, respectively). Patients with middle and high SES were more likely to complete the test-pathway compared to patients with low SES (OR 1.71, 95% CI 1.10–2.65, *p* = 0.016; OR 1.95, 95% CI 1.15–3.31, *p* = 0.013, respectively). Finally, patients who received surgery were nine times more likely to complete the test-pathway compared to patients who did not receive surgery (OR 9.66, 95% CI 6.07–15.40, *p* < 0.001) and patients who received chemotherapy were four times more likely to complete the test-pathway compared to those who did not receive chemotherapy (OR 4.07, 95% CI 2.73–6.05, *p* < 0.001).

**Table 3:** Predictors of completing the *BRCA1/2* test-pathway in patients with epithelial ovarian cancer

	Categories	*n*	Univariate			Multivariable		
			OR	95% CI	*p*-value	OR	95% CI	*p*-value
Age at diagnosis, per 5 years		1085	0.75	0.70–0.79	< 0.001	0.89	0.83–0.96	0.002
Year of diagnosis, in TF years	First year	269	1		0.004	1		< 0.001
	Second year	293	1.12	0.79–1.59	0.512	1.53	0.96–2.44	0.077
	Third year	273	1.76	1.22–2.55	0.003	2.52	1.52–4.20	< 0.001
	Fourth year	250	1.67	1.14–2.43	0.008	3.65	2.11–6.30	< 0.001
Region	A	568	1					
	B	517	1.04	0.80–1.35	0.762			
FIGO stage	I/II	247	1		< 0.001	1		0.016
	III/IV	816	1.20	0.88–1.63	0.250	2.10	1.24–3.53	0.005
	Unknown	22	0.07	0.02–0.26	< 0.001	0.91	0.18–4.50	0.908
Histotype	Other	447	1			1		
	HGS/HGEn	638	4.48	3.40–5.91	< 0.001	2.86	1.96–4.18	< 0.001
SES	Low	242	1		< 0.001	1		0.044
	Middle	482	1.91	1.39–2.64	< 0.001	1.71	1.10–2.65	0.016
	High	241	2.86	1.91–4.26	< 0.001	1.95	1.15–3.31	0.013
	Unclassifiable	120	1.94	1.12–3.11	0.006	1.30	0.69–2.43	0.420
Performance status	0 to 1	478	4.09	2.58–6.48	< 0.001	1.45	0.78–2.69	0.244
	2 or higher	98	1		< 0.001	1		0.300
	Unknown	509	1.40	0.91–2.16	0.129	1.06	0.57–1.98	0.854
Comorbidities	0	352	2.78	1.11–6.95	0.029	1.40	0.38–5.16	0.613
	1 to 2	206	1.82	0.72–4.60	0.208	1.60	0.43–6.01	0.486
	≥ 3	20	1		0.001	1		0.871
	Unknown	507	1.55	0.63–3.82	0.338	1.37	0.37–5.04	0.637
Surgery	No	341	1			1		
	Yes	744	10.22	7.57–13.79	< 0.001	9.66	6.07–15.40	< 0.001
Chemotherapy	No	341	1			1		
	Yes	744	10.22	7.57–13.79	< 0.001	4.07	2.73–6.05	< 0.001

Abbreviations: OR, odds ratio; CI, confidence interval; TF, tumor-first; FIGO, Fédération Internationale de Gynécologie et d’Obstétrique;HGS, high-grade serous; HGEn, high-grade endometrioid; SES, socio-economic status

## Discussion

This study shows that, in a real-world clinical setting with a nationwide implemented tumor-first testing workflow, approximately one out of three EOC patients did not receive the recommended *BRCA1/2* testing. The overall percentage of patients who completed the test-pathway did significantly increase in the third and fourth year compared to the start, with 74.4% completing the test-pathway in the most recent year. Patients were also significantly more likely to complete the test-pathway if they were younger at diagnosis, diagnosed with high-grade serous/high-grade endometrioid carcinoma, diagnosed with stage III/IV, had high or middle SES, and had received chemotherapy or surgery.

A tumor testing workflow would, theoretically, result in greater equity with respect to access to DNA testing in comparison to former germline-based workflows. Significant improvements in testing rates were reported in patients with high-grade serous ovarian cancer after implementation of reflex *BRCA1/2* tumor testing [18]. Furthermore, preliminary results of the nationwide implementation project showed that tumor tests were offered to more than 80% of all included patients [8]. Here, we took a rigorous patient-centered approach to evaluate full completion of the tumor-first test-pathway, and our evaluation shows that inequities in testing still remain. Clinical and patient characteristics independently influence the likelihood of completing the test-pathway, and these insights may be used to address barriers to testing, as outlined below.

First, a younger age and high-grade serous/high-grade endometrioid histotype were significantly associated with higher odds of completing the test-pathway. This finding may originate from the clinician’s perspectives that patients with these characteristics are more likely to carry a PV. Nevertheless, the mean age of diagnosis in *BRCA2* carriers does not substantially differ from that in sporadic EOC diagnoses [19, 20]. Additionally, while we have previously shown that causal *BRCA1/2* PVs were exclusively detected in high-grade serous/high-grade endometrioid carcinoma [17], selectively testing these tumors requires accurate histopathological diagnosis and possibly central pathology reviews, which is not structurally implemented before testing. To optimize identification of potential *BRCA1/2* carriers in this patient population, it is advised to test all patients regardless of their age and histotype [7].

Next, patients with advanced-stage disease were significantly more likely to complete the test-pathway compared to those with early-stage disease. This could be driven by the PARP inhibitor indication being limited to patients with a PV in the tumor and advanced-stage disease in the first-line setting [21]. However, the tumor test is not solely implemented to determine PARP inhibitor sensitivity, but simultaneously determines potential genetic predisposition. Therefore, testing patients with early-stage disease remains essential to determine genetic predisposition and subsequently reduce cancer incidence and mortality among relatives of the patient.

Importantly, patients with high and middle SES were approximately two times more likely to complete the test-pathway compared to patients with low SES. Considering the pathology-based universal reflex testing and full reimbursement of tests, we anticipated the influence of SES on the likelihood of receiving the test to be limited. However, low SES has been linked to less aggressive treatment strategies and lower survival rates in ovarian cancer patients [22], which could explain the observed inequities. Regardless of SES, patients who received chemotherapy or surgery were more likely to complete the test-pathway compared to patients who did not receive chemotherapy or surgery. While chemotherapy and surgery are standard of care for patients with FIGO stage IIb-IV, 31% of the patients in our cohort did not receive chemotherapy and 31% did not receive surgery. Not receiving chemotherapy could indicate a rapidly progressing disease, where chemotherapy could not be timely provided or was not expected to provide benefits. Not receiving surgery limits the availability of tumor tissue required for the tumor test. DNA can also be isolated from ascites or tissue obtained by biopsies, but this could not be evaluated in the current study. It is essential that clinicians are aware of the need to refer patients for a germline test, even when prognosis is poor or tumor tissue is not available, as determining genetic predisposition can have lifesaving consequences for relatives.

Not all patients with a positive tumor test and subsequent referral to clinical genetic services (i.e., completion of the test-pathway) actually proceeded with germline testing. Notably, patients may decline a germline test. Therefore, we decided to analyze the percentage of patients referred rather than tested, as this most accurately reflects the extent of adequate test-pathway implementation. In our series, a few patients passed away after referral and before the germline test, highlighting that lack of time due to poor prognosis potentially limits test-pathway completion. Implementing mainstream germline testing within the tumor-first pathway could accelerate the process of determining genetic predisposition and further increase test uptake [23]. This would be particularly valuable for patients with rapidly progressing disease and for the relatives of these patients. Under a mainstream germline testing pathway, pre-test genetic counseling and blood sampling would be provided by trained gynecologic oncologists as part of routine care. Post-test counseling with a clinical genetics service would be offered to those who carry a PV, as well as to patients without a PV, but with a relevant personal or family history (e.g., personal history of breast cancer, or personal/family history of Lynch syndrome-associated cancer). Nevertheless, the high morbidity and mortality of EOC patients complicate testing uptakes, emphasizing the need to adequately counsel relatives if the patient cannot be tested.

A limitation of this study is that we heavily relied on reported data in patient files. While it is not expected that patients were genetically tested without this being reported, it is unknown to what extent tests performed outside of our regions, relocation of patients, declining a test, or testing of family members, are being reported in patient files. Our estimates are, therefore, a real-world reflection of what is reported in patient files but could underestimate the actual uptake. Regional hospitals were contacted to obtain additional information on untested patients. Moreover, we acknowledge that a tumor- rather than germline-based testing approach is not endorsed by all countries. Yet, our study approach and the identified patient populations that were less likely to be tested are informative and likely applicable to other (germline-based) testing approaches.

In conclusion, approximately one out of three EOC patients was not offered *BRCA1/2* testing. This study highlights the need for better adherence to current guidelines, particularly in older patients, those with low SES, low-grade histotypes, early-stage disease and those who do not undergo surgery or chemotherapy. Additionally, timely DNA testing, and testing relatives if testing the patient is not an option are crucial to increase testing rates. Ultimately, this will enhance care for patients and reduce the incidence and mortality of cancer in relatives with a hereditary predisposition for EOC.

## Electronic supplementary material

Below is the link to the electronic supplementary material.

Supplementary Material 1

## Data Availability

The data underlying this article will be shared on reasonable request.
