# Supplementary material for: Evaluation of BRCA1/2 testing rates in epithelial ovarian cancer patients: lessons learned from real-world clinical data
Source: Fam Cancer. 2025 May 5;24(2):43. doi: 10.1007/s10689-025-00467-7 (PMC12053180; doi:10.1007/s10689-025-00467-7)
Supplement: Supplementary file 1 — Supplementary Material 1 [file 10689_2025_467_MOESM1_ESM.docx]

**Supplementary materials**

Supplementary Table 1. Variable descriptions.

| **Variables** | **Descriptions** |
| --- | --- |
| Date of diagnosis | Defined as date of first histological or cytological confirmation of tumor. |
| Year of diagnosis | Date of diagnosis classified into years of tumor-first approach: first, second, third or fourth year. |
| Age of diagnosis | Defined as the time interval (in years) between date of birth and date of diagnosis. For the regression analysis, this variable was expressed per 5 years. |
| Region of diagnosis | Catchment areas of two specialized gynecologic oncology university hospitals, classified as A or B. |
| Histotype | Based on the World Health Organization (WHO) International Classification of Diseases for Oncology (ICD-O), Third Edition. This includes the update ICD-O-3.2 for diagnoses from 2020 onwards. Classified into high-grade serous, endometrioid, low-grade serous, clear cell, mucinous, carcinosarcoma, other, and adenocarcinoma not otherwise specified (NOS) by an expert gynecopathologist. For the regression analysis, high-grade serous carcinomas (*n*=612) and high-grade endometrioid carcinomas (*n*=26) were combined and compared to other histotypes. |
| FIGO stage | Classified as stages I, II, III, IV and unknown. |
| Performance status | Evaluated according to the WHO performance status scoring system. Assessed before start of treatment as asymptomatic (0), symptomatic but completely ambulatory (1), symptomatic, <50% in bed during the day (2), symptomatic, >50% in bed, but not bedbound (3), bedbound (4), or death (5). |
| Comorbidities | Evaluated according to the weighted score of the Charlson Comorbidities Index. Includes comorbidities that were present at time of diagnosis or developed after diagnosis but before start of treatment and influenced the treatment plan. |
| Socioeconomic status | Based on full postal codes that cover both demographic and socioeconomic data. Data was available in 10 categories: low (1), low to below mid (2), below mid (3), below mid to mid (4), mid (5), mid to above mid (6), above mid (7), above mid to high (8), high (9), unclassifiable (10), and merged into low socioeconomic status (1-3), middle socioeconomic status (4-6), high socioeconomic status (7-9), and unclassifiable. The unclassifiable category includes postal codes with very few or no houses, including industrial and rural areas. |
| Receival of surgery | Defined as the receival of diagnostic laparoscopy, primary debulking or interval debulking following the diagnosis of epithelial ovarian cancer (yes/no). |
| Receival of chemotherapy | Defined as the receival of at least 1 cycle of chemotherapy following the diagnosis of epithelial ovarian cancer (yes/no). |


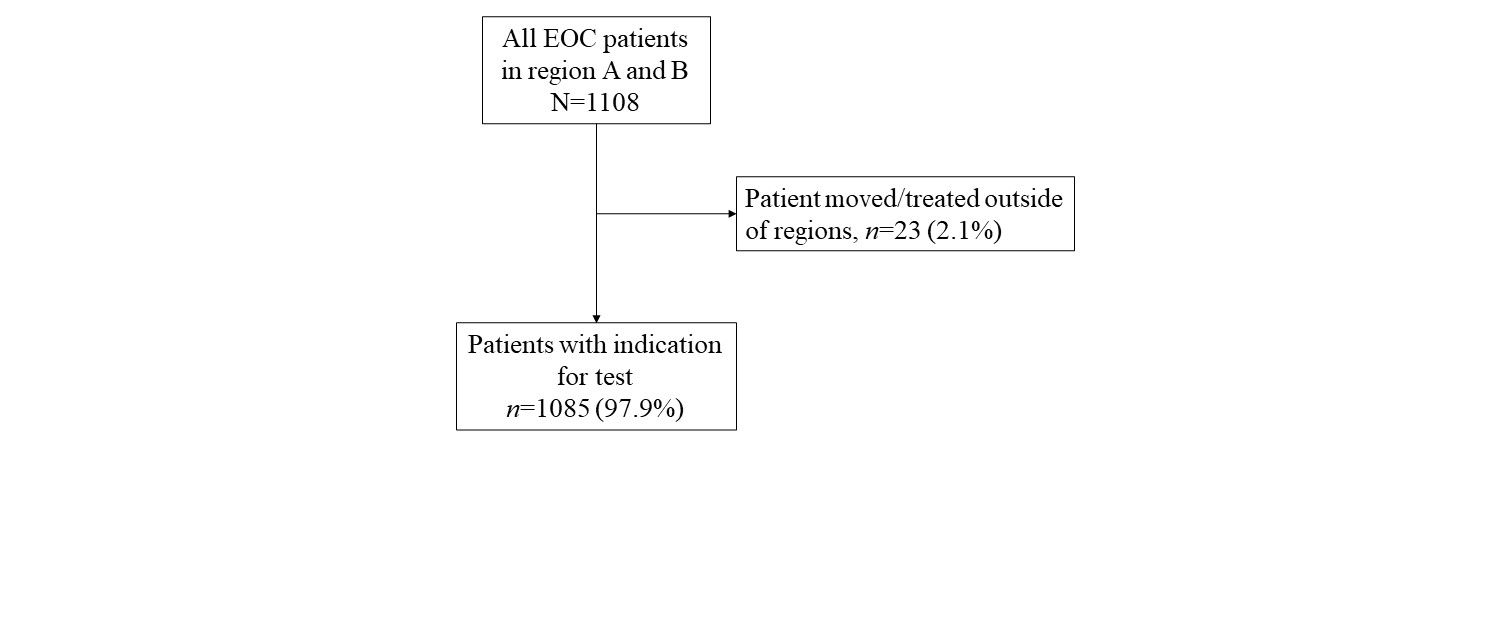


Supplementary Figure 1. Flowchart of patients with indication for *BRCA1/2* test.

Abbreviations. EOC, epithelial ovarian cancer.

Supplementary Table 2. Outcomes of the *BRCA1/2* test-pathway evaluation in patients with indication for test for region A and B, *n* (%).

| **Characteristics** | **Patients with indication for test**  **(*n*=1085)** | **Region A**  **(*n*=568)** | **Region B**  **(*n*=517)** |
| --- | --- | --- | --- |
| Completed test-pathway^1^ | 757 (69.8) | 394 (69.4) | 363 (70.2) |
| Known germline status | 743 (68.5) | 388 (68.3) | 355 (68.7) |

^1^ Includes 3 patients who passed away between referral and germline test.
